# Neogenin expression may be inversely correlated to the tumorigenicity of human breast cancer

**DOI:** 10.1186/1471-2407-5-154

**Published:** 2005-12-03

**Authors:** Jeong Eon Lee, Hee Joung Kim, Ji Yeon Bae, Seok Won Kim, Joon-Suk Park, Hyuk Jai Shin, Wonshik Han, Sung-Won Kim, Kyung-Sun Kang, Dong-Young Noh

**Affiliations:** 1 Department of Surgery, Seoul National University College of Medicine, 28 Yongon-dong, Chongno-gu, Seoul 110-744, Korea; 2 Cancer Research Institute, Seoul National University College of Medicine, 28 Yongon-dong, Chongno-gu, Seoul 110-744, Korea; 3 Department of Surgery, Eulji University Hospital, 1306 Dunsan-dong, Seo-gu, Daejeon 302-799, Korea; 4 Laboratory of Stem Cell and Tumor Biology, Department of Veterinary Public Health, College of Veterinary Medicine, Seoul National University, San 56-1, Shilim-dong, Kwanak-gu, Seoul 151-742, Korea; 5 Department of Surgery Seoul National University Bundang Hospital, 300 Gumi-dong, Bundang-gu, Seongnam-Si, Gyeonggi-do 463-707, Korea

## Abstract

**Background:**

Neogenin is expressed in cap cells that have been suggested to be mammary stem or precursor cells. Neogenin is known to play an important role in mammary morphogenesis; however its relationship to tumorigenesis remains to be elucidated.

**Methods:**

To compare the expression levels of neogenin in cells with different tumorigenicity, the expression levels in M13SV1, M13SV1R2 and M13SV1R2N1 cells, which are immortalized derivatives of type I human breast epithelial cells, were evaluated. Then we measured the expression level of neogenin in paired normal and cancer tissues from eight breast cancer patients. Tissue array analysis was performed for 54 human breast tissue samples with different histology, and the results were divided into four categories (none, weak, moderate, strong) by a single well-trained blinded pathologist and statistically analyzed.

**Results:**

The nontumorigenic M13SV1 cells and normal tissues showed stronger expression of neogenin than the M13SV1R2N1 cells and the paired cancer tissues. In the tissue array, all (8/8) of the normal breast tissues showed strong neogenin expression, while 93.5% (43/46) of breast cancer tissues had either no expression or only moderate levels of neogenin expression. There was a significant difference, in the expression level of neogenin, in comparisons between normal and infiltrating ductal carcinoma (p < 0.001).

**Conclusion:**

Neogenin may play a role in mammary carcinogenesis as well as morphogenesis, and the expression may be inversely correlated with mammary carcinogenicity. The value of neogenin as a potential prognostic factor needs further evaluation.

## Background

Neogenin, a member of the DCC (Deleted in Colorectal Cancer) receptor group, encodes a 1461 amino acid protein and has similar structure to DCC with 50.2% amino acid identity [1]. Neogenin is one of two groups of netrin-1 receptors [1,2]. Netrin-1 and its receptor groups were initially known to induce neurons and axons to target certain areas during neurogenesis [3]. Netrin-1 and neogenin do not only regulate axonal guidance but also play an important role in epithelial morphogenesis [4]. Neogenin is a cell adhesion molecule, and is expressed in a variety of developing tissues, such as the gastrointestinal tract of chickens [5], as well as on the surface of growing neurons [6].

Neogenin has been found to be expressed in cap cells at the terminal end buds of breast tissue. Cap cells have been suggested to be mammary stem or precursor cells located in the terminal ductal lobular units of the breast [7,8]. Recent data show that netrin-1 and its receptor neogenin provide an adhesive function during mammary gland morphogenesis; the binding of netrin-1 and neogenin stabilize the cap cell layer and prevent the influx of cap cells into the prelumenal compartment; this keeps the cap cells in their initial location [9]. The relationship between the expression of neogenin and the tumorigenicity of breast cancer has not been studied to date.

Kao *et al. *reported on obtaining two types of human breast epithelial cells (HBECs) in a breast tissue specimen from reduction mammoplasty [10]. Type I HBECs are known as potent breast stem cell candidates; they can differentiate into type II HBECs, and form budding/ductal organoids in Matrigel. It is also reported that type I HBECs have luminal epithelial cell markers, and stem cell characteristics, and that type II HBECs show basal epithelial phenotypes [11,12]. Nontumorigenic M13SV1 cells are immortalized cells obtained from the transfection of type I HBECs with simian virus 40 large T antigens. M13SV1R2 cells, obtained after x-ray irradiation (two doses of 2 Gy 8 days apart) of M13SV1 cells, express weak tumorigenicity when they are injected into athymic nude mice. The highly tumorigenic M13SV1R2N1 cells result from transduction of the rat *neu *oncogene into M13SV1R2 cells [11,13].

We hypothesized that neogenin might be related to mammary carcinogenesis. Therefore, we evaluated expression levels of neogenin in the M13SV1 cell line series (M13SV1, M13SV1R2, and M13SV1R2N1), the immortalized tumorigenic derivatives from type I HBECs, to compare the expression of neogenin in cells with different tumorigenicity. Then, we investigated neogenin expression in paired normal and cancer tissues from eight patients with infiltrating ductal cancer. Finally, to identify the pattern of neogenin expression on the basis of different histology, we performed tissue array analysis of 54 different human breast tissue samples.

## Methods

### Cells, cell culture and tissues

The M13SV1, M13SV1R2 and M13SV1R2N1 cells were cultured in MSU-1 medium, with 10% fetal bovine serum, containing 100 units of penicillin and 100 μg of streptomycin/ml, in a humidified incubator at 37°C with 5% CO_2 _[13].

To determine the expression levels of neogenin, in paired normal and cancer tissues, from the same patients, we selected paired specimens from eight patients and stored the samples in a liquid nitrogen tank. All of the patients had the diagnosis of an infiltrating ductal carcinoma. Normal tissues were sampled and were obtained from sites more than 5 cm away from the tumor margin of the cancer. All of the tissues were obtained and stored with the consent of each patient. The tissues to be used for the tissue array were stored in paraffin-embedded blocks.

### Western blot analysis

The M13SV1, M13SV1R2 and M13SV1R2N1 cells were lysed in 20 mM HEPES (pH 7.2), 150 mM NaCl, 1% Triton X-100, 2% cholic acid, 1 mM EDTA, 1 mM EGTA, 0.1 mM DTT, 10 μg/ml leupetin, 10 μg/ml aprotinine and 1 mM phenylmetylsulfonyl fluoride (PMSF). Protein content (25 μg) was determined by the bicinchoninic acid (BCA) protein assay with bovine serum albumin as a standard. Cell lysates were released by heating samples at 95°C for 10 minutes in Laemmli cooking buffer and then separated by SDS (sodium dodecylsuldate)-polyacrylamide gel electrophoresis. Samples were transferred onto nitrocellulose filters and incubated separately with primary antibodies for two hours. Immunocomplexes were visualized using the peroxidase conjugated goat anti-rabbit IgG antibody. Detection was performed with the ECL Plex fluorescent Western blotting system (Amersham, Buckinghamshire, UK). The paired cancer and normal tissues from eight patients were treated in the same way. We have examined the expression of neogenin with 1:200 diluted anti-neogenin rabbit IgG polyclonal antibody (#sc-15337, Santa Cruz Biotechnology, California) as a primary antibody, and 1:5000 diluted horseradish peroxidase conjugated goat anti-rabbit IgG antibody (#sc-2004, Santa Cruz Biotechnology, California) as a secondary antibody.

### Tissue array

Tissue array analysis was performed to examine the expression of neogenin in a variety of tissue types under the same conditions. We tested the tissues from the following sources: 8 normal, 31 infiltrating ductal carcinomas (IDC), 1 ductal carcinoma in situ (DCIS), 9 metastatic lymph nodes from breast cancer, and 5 other types of breast cancer cases (3 cases of invasive lobular carcinoma (ILC), one medullary carcinoma and one signet ring cell carcinoma). Tissues were embedded in paraffin; 4-μm sections, stained with hematoxylin and eosin (H&E), were obtained to identify morphologically representative areas of the specimen from which the core biopsies were taken. We marked the sampling site, on the corresponding blocks, with H&E stained slides. The blank paraffin blocks were then punched with a precision instrument (SuperBioChips Laboratory, South Korea), to make 2 mm × 60 holes; we then punched out the marked area of donor blocks, and filled in the holes of the recipient block. In this way, triplicate tissue cores, with diameters of 2 mm, were punched and arrayed on a recipient paraffin block. A layer of paraffin was coated to complete an array block with tightening of the paraffin. Then arrayed paraffin blocks were cut into 4-μm sections and placed on silane coated slides (MUTO pure chemicals, Tokyo, Japan). Sections from the paraffin-embedded tissue were deparaffinized, treated with 1% H_2_O_2 _in phosphate-buffered saline, and submitted for antigen retrieval by microwave oven treatment for 15 minutes in 0.01 mmol/L citrate buffers at pH 6.0. The slides were subsequently incubated in 10% normal horse serum for 30 minutes, followed by overnight incubation, at 4°C, with appropriately diluted rabbit polyclonal IgG antibodies to neogenin as a primary antibody and the horse radish peroxidase conjugated goat anti-rabbit IgG antibody as a secondary antibody. Fixation control was not used. Antibodies were the same as those used for the Western blot analysis, and diamino-benzidine (DAB) was used as a chromogen.

### Statistical analysis

Fisher's exact test from the SAS statistical software Version 8 program (SAS Institute Inc., Cary, NC) was used for statistical calculation. All statistical analyses were two-tailed. We confirmed that all statistical results were reproducible with SPSS Version 11.5 (SPSS Inc., Chicago, IL).

## Results

**Figure 1:**
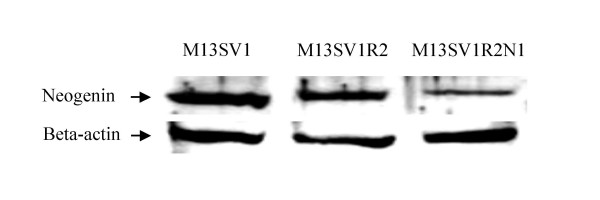
The Western blot analyses of M13SV1, M13SV1R2 and M13SV1R2N1 cells. The expression of neogenin is strong in nontumorigenic M13SV1 cells while it is weak in highly tumorigenic M13SV1R2N1 cells. Detection is performed with the ECL Plex fluorescent Western blotting system (Amersham, Buckinghamshire, UK).

**Figure 2:**
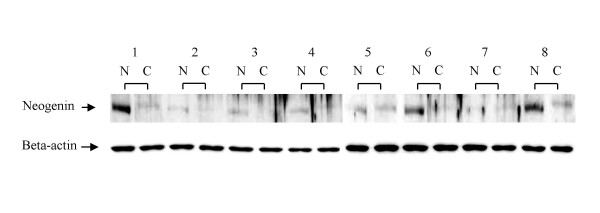
Western blot analyses of paired normal and cancer tissues from eight patients with infiltrating ductal carcinoma. There is a tendency toward higher neogenin expression in normal tissues compared to the IDC tissues; this is clear in samples 1–4, 6 and 8. Detection is performed with the ECL Plex fluorescent Western blotting system (Amersham, Buckinghamshire, UK).

As a result of the Western blot analysis from the three cell lines, the data showed that the expression of neogenin was strongest in the nontumorigenic M13SV1 cells. Conversely, in the highly tumorigenic M13SV1R2N1 cells, neogenin was found to be only weakly expressed (Fig. 1). Western blot analysis of paired normal and IDC tissues from eight patients with breast cancer showed that the strength of expression of neogenin in normal tissue varied in each patient. However, there was a tendency toward higher expression of neogenin in normal tissues compared to the IDC tissues; this was clearly observed in 6 of 8 paired samples (Fig. 2).

**Figure 3:**
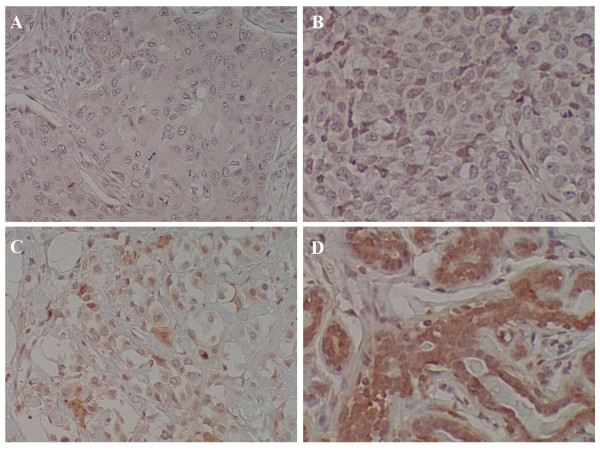
The tissue array evaluated by a single pathologist showed different levels of neogenin expression in the various tissue samples. (A) There is no expression of neogenin observed in an IDC sample. (B) Another IDC sample shows a weak level of expression. (C) There is a moderate level of neogenin expression in an infiltrating lobular carcinoma sample. (D) A strong expression level of neogenin is observed in normal tissue. All pictures were magnified as × 200.

Tissue array analysis was performed on 54 diverse human breast tissue samples with different histologic diagnoses (Table 1). The expression levels were measured, by a single blinded senior pathologist, based on the number of positive cells and the staining intensity; there were four categories: none, weak, moderate and strong, used for classification (Fig. 3). In concordance with the results of Western blot analysis, all of the normal breast tissues showed strong neogenin expression, while 93.5% (43/46) of breast cancer tissues showed no neogenin expression or only moderate levels. In 31 IDC cases, five (16.1%) did not have any neogenin expression, fourteen (45.2%) showed weak expression, ten (32.3%) showed moderate expression, and only two (6.5%) had strong levels of neogenin expression. There was a significant difference (p < 0.001) in the expression level of neogenin in normal compared to IDC cases.

## Discussion

The results of the present study suggest that the expression of neogenin may be inversely related to the tumorigenicity of human breast cancer. In the Western blot analysis, neogenin expression is noted to be strong in nontumorigenic M13SV1 cells and decreased as the tumorigenicity increased; neogenin expression was weakest in the highly tumorigenic M13SV1R2N1 cells. The paired Western blot analysis from normal and IDC tissues in eight patients also resulted in an inverse pattern of neogenin expression with cancer. In the tissue array, all of the normal breast tissues showed a strong neogenin expression, while most cancer tissues showed weaker neogenin expression compared to normal tissues. These results suggest that the loss of neogenin expression might be important in mammary carcinogenesis.

Human mammary glands begin to develop with the primitive mammary epithelial cells growing into epithelium during the sixth week of fetal life. During the first three weeks of human life, the rudimentary mammary duct consists of a single duct gradually extending from the nipple. By the fifth week of age, the ducts fill approximately two-thirds of the fat pad, with a highly branched system that exhibits second and third order branching [14]. The terminal end bud (TEB) is the enlarged termini of ducts; a single layer of cap cells covers the tip of the end bud. TEBs are responsible for prodigious pubertal growth of cap cells and underlying preluminal epithelium. Cap cells have been suggested as mammary stem cells for ductal morphogenesis [7] or as cells serving as precursors to myoepithelial cells [8]. During puberty, the cap cells begin to grow into the adjacent fatty tissue and the parenchyma gains more fatty tissue and vessels. The accurate approximation of cap cells and preluminal epithelial cell layers is thought to be important for mammary morphogenesis; netrin-1 and neogenin play an important role in maintaining this approximation. The loss of neogenin during murine mammary morphogenesis results in widening of the subcapsular space; this anatomical detachment causes dissociation of the cap cells, and increased spillage of cap cells into the preluminal compartment. This may contribute to susceptibility for the development of human breast cancer [9,15].

Although a previous study reported that neogenin was expressed in breast cancer cell lines, and suggested that neogenin is not likely to be frequently affected in cancer, this study lacked important data at the translational level for the expression of neogenin in normal and breast cancer tissues [1]. Consistent with previous reports, neogenin was expressed in breast cancer cell lines in the present study. Moreover, we identified that there was a difference in the expression level of neogenin based on the tumorigenicity; this was observed as a result of experiments with three cell lines that have the same origin as the type I HBECs but different tumorigenicity. In the Western blot analysis for paired specimens, neogenin showed stronger expression in normal tissues than in each of the paired cancer samples.

It was difficult to determine the exact intensity of neogenin expression in tissue arrays with anti-neogenin polyclonal antibody. We could not find any commercial monoclonal anti-neogenin antibody, only polyclonal antibody. Future experiments with more specific monoclonal antibodies will be required for the precise demonstration of neogenin expression in tissues by immunohistochemical stain.

## Conclusion

In summary, neogenin may play a role in mammary carcinogenesis as well as in mammary morphogenesis, and the expression of neogenin may be inversely correlated to mammary carcinogenicity. Further investigation is needed to identify the value of neogenin as a potential prognostic factor.

## Competing interests

The author(s) declare that they have no competing interests.

## Authors' contributions

All the authors contributed to the conception during the initial stages and study design, and in the analysis and interpretation of the data, as well as to the drafting and critical revision of the important intellectual content. All the authors agreed to the final approval of the version to be published. JEL and HJK were equally involved in this study. JYB performed the Western analyses. DYN was in charge of the general supervision of this research. The coauthors declare the order of authorship was based on a joint decision.

## Pre-publication history

The pre-publication history for this paper can be accessed here:



## Figures and Tables

**Table 1:** The distribution of neogenin expression according to the histology in the tissue array

Histology	Expression level of neogenin				Total
		
	None	Weak	Moderate	Strong	
Normal	0	0	0	8	8
Ductal carcinoma in situ	0	0	1	0	1
Infiltrating ductal carcinoma	5	14	10	2	31
Metastatic lymph node	2	3	4	0	9
Others*	0	1	3	1	5
Total	7	18	18	11	54

*Others consist of 3 cases of invasive lobular carcinoma, 1 medullary carcinoma and 1 signet ring cell carcinoma.
